# Ethical Challenges of Cross-Cultural Research – The Example of a Psychological Research Project in the Andean Context

**DOI:** 10.1177/15562646231181880

**Published:** 2023-06-20

**Authors:** Helen Wefers, Vanessa Krüger, Nancy Beatriz Iza Simba, Yuri Amaya Guandinango

**Affiliations:** 1 9185 University of Münster, Germany; 2 9186 University of Osnabrück, Germany; 3 216226 University of Otavalo, Ecuador; 4 185009 Facultad Latinoamericana de Ciencias Sociales, Ecuador

**Keywords:** cross-cultural psychology, ethics, research relationships, horizontality

## Abstract

Using the example of a psychological research project in the Andean context, this explorative interview study adds to the current debate about ethical challenges of psychological cross-cultural research projects by being one of the first to address those challenges empirically. Using a multilevel approach, we conducted interviews with three groups of experts (study participants, i.e., Kichwa mothers, research assistants and experts on research in Indigenous communities in Ecuador; *N* = 10). Overall, the thematic analysis identified that the establishment of horizontal research relationships allows the best adaption to context-specific values (e.g., balance between giving and receiving), norms and societal structures. On the grounds of the analysis, we derived reflective questions for addressing the complex ethical challenges in future cross-cultural projects.

The scientific knowledge and concepts generated in developmental psychology (Nielsen et al., 2017) and adjacent fields (Henrich et al., 2010) are largely based on samples unrepresentative of the world population. Despite discussions about how to increase the inclusivity, representativeness and realism of cross-cultural research, Nielsen et al. (2017) pointed to the continuing trend that “the vast majority of the world's population is underrepresented in high impact developmental psychology research” (Nielsen et al., 2017, p. 34). Thus, from a scientific perspective and from the specific perspective of cross-cultural developmental research, the empirical basis urgently needs diversification.

Along with this skew in sampling, theoretical foundations and research methodology from the Global South remain excluded from scientific discourses; specifically, researchers studying WEIRD samples (Western, educated samples from industrialized, rich and democratic countries) continue to create representations of the cultural *Other*. From a postcolonial viewpoint, those patterns of power (e.g., to define knowledge: Who defines the criteria for scientific performance?) and privilege (Who is researched by whom, by what right and who profits from the findings?) are historically rooted and are symptomatic of the dominance of Western epistemology (Bhatia, 2018; Mignolo, 2002; Smith, 2021).

As such, along with the abovementioned argument for expanding cross-cultural studies, we note in accordance with Broesch et al. (2020) (see also Bruno et al., 2022) that these studies are associated with unique ethical challenges. Some such challenges arise from implicit or explicit power differentials between the researchers and the researched that are created and reproduced in the context of many cross-cultural studies taking place in former or contemporary colonies. To our knowledge, the ethical challenges of psychological cross-cultural research projects that occur in (post)colonial contexts have not yet been empirically investigated. However, in light of the ethical challenges of those projects, there is an urgent need to know more about the perceptions of study participants and about conceptual (epistemological, methodological, and regarding the design of research relationships) solutions to context-specific ethical challenges. This need is further exemplified by current debates about racism and racial justice (e.g., the Black Lives Matter movement), which, according to Urassa et al. (2021), have the positive impact of “reinvigorating efforts to decolonize the social sciences” (p. 668).

Against this background, this explorative interview study has three aims: First, it aims to study ethical conduct (perceptions of participants regarding research processes and implementation; e.g., balance between giving and receiving) using the example of a developmental psychology research project on early mother-infant interaction occurring from 2017 to 2018 in communities around Cotacachi and Otavalo in Ecuador (overall project). Second, it aims to address the question of how research between equal partners can succeed in *this* cultural context. Third, it aims to establish educational measures – for example, trainings with the whole research team – that help achieve research relationships based on partnership. Each of us (the authors) contributed to the realization of the overall project: Helen Wefers initiated and led the project and conceptualized the project together with Yuri Amaya Guandinango; Vanessa Krüger and Nancy Beatriz Iza Simba helped coordinate and realize the data assessments in Ecuador.

## The Power to Define: Power Asymmetries and the Production of Knowledge

For decades, academics have reflected on the connection between knowledge production and social constellations of power (e.g., Maldonado-Torres, 2007; Mignolo, 2002). Looking at Euro-American psychology from a decolonial perspective, Bhatia (2018) calls attention to the discipline's colonizing effects in the past and present. For example, criteria for scientific performance (e.g., quality criteria in quantitative and qualitative research; randomized controlled trails as the methodological gold standard) are defined by researchers from the Global North, and meeting those criteria is a prerequisite for publishing in high-ranking journals. Further critical positions on power asymmetries within the field of psychology have recently been formulated by researchers conducting cross-cultural studies, some of which call for horizontal forms of knowledge production such as community-based participatory research and mixed methods in data collection (with the aim of converging evidence; e.g., Broesch et al., 2020).

### Ethical Challenges in Research Projects Conducted by Global North Researchers in Global South Contexts^i^

In the following, we argue that relationships and processes in cross-cultural research projects that take place in former colonies continue to be characterized by power asymmetries (e.g., between researchers and participants/research assistants/institutions). In recognition of colonial history and its potential consequences (e.g., for knowledge production) at present day, we view those asymmetries as potentially unethical, for example, (a) when one's concept of the other person is characterized by external ascriptions (Riaño, 2012), e.g., homogenously viewing the other as underdeveloped, exotic, without appropriate expertise, (b) when local knowledge is marginalized, when (c) the power to define (Riaño, 2012, e.g., the goals of the project, the work steps, the units for analysis) or (d) the profits from the findings (e.g., regarding increased knowledge, international recognition or publications) are unequally distributed.

Another unique challenge is that researchers and the researched in cross-cultural studies typically differ in cultural socialization, namely in their cultural values and norms. Yet, a deep understanding of and respect for the culture is a prerequisite for ethical conduct, because universal ethical guidelines need to be translated in a culture-sensitive way.

## Toward a Globally Representative and Inclusive Developmental Science

If psychological cross-cultural research projects are so ethically challenging, why would one argue for expanding those studies in, for example, the field of developmental psychology? Along with other researchers, we stress that globally representative sampling is required for understanding both universal and cultural variation in child development, because “there is no universal developmental context in which children grow up, nor is there a universal for the human mind” (Nielsen et al., 2017, p. 32).

While many psychologists conducting cross-cultural research criticize the skew toward WEIRD sampling, only few call for and implement both a diversification of databases and a diversification of scientific perspectives. From our viewpoint, different systems of knowledge within developmental science urgently need to communicate, for ethical reasons and for reasons of quality (e.g., to ensure a shared meaning across measured constructs, Marfo, 2016).

## Case Study: A Psychological Research Project in the Andean Context

Exploring infant development cross-culturally by drawing on quantitative research methodology, Helen Wefers, doctoral student at the University of Münster (Germany), initiated a developmental psychology research project with data assessments from 2017 to 2018 in Germany and Ecuador (overall project). As the German research team had a greater power to define (research objectives and methods) than the local Ecuadorian research team, this overall project left unrecognized large parts of the abovementioned critique. The overall project had a longitudinal research design and focused on implicit maternal ideas about ideal states of infant affect and activity (Wefers et al., 2022), mother-infant interaction around infant smiling (Kärtner et al., 2022), and early social expectations (Wefers et al., 2023). The project involved weekly visits from postnatal week 8 to 18. In Ecuador, 31 mothers who identified as Kichwas participated with their babies. They lived in communities in the surroundings of Cotacachi or Otavalo (canton Cotacachi). In total, ∼20 assistants (students or employees) from the University of Otavalo (Ecuador) and the University of Münster (Germany) conducted the assessments. More precisely, bicultural teams of two assistants visited the same family 10–12 times for assessments. Most of the assistants from Ecuador identified as Kichwas; two students identified as Mestizxs.

Viewing the data assessment in rural Ecuador from a power-critical viewpoint (Kaltmeier & Berkin, 2012), a clear hierarchy between researchers and the researched could be observed. Within those hierarchical structures, the German-Ecuadorian research team established some reciprocal elements aiming to respond to ethical challenges in a culture-sensitive way and to involve the community in the research processes and in the returning of research results. On the one hand, the research team members from Ecuador had explicitly asked the project leader to what extent she was giving something back to the target group. At the same time (from a retrospective view), the historical parallel regarding power asymmetries troubled the project leader considerably during the whole process, and it was partly from this discomfort that we developed those reciprocal elements. Along with material (e.g., alimentation, namely rice, sugar and oats) and financial compensation for participating families, the German-Ecuadorian research team developed workshops about child development (e.g., motor development, language, healthy alimentation) and a safe home; to develop the workshops, the team drew on local knowledge and aimed at strengthening mothers in their roles. During the main assessments, we asked mothers what kind of workshops they would be interested in and prepared them accordingly. We also had a closing event with families and student assistants where we presented and discussed initial results.

Once the data assessment of the overall project in Ecuador had ended, the first authors (both socialized in the Global North) conducted this explorative interview study because they were interested in the participating mothers’ perceptions of this project and the reciprocal elements were applied. That is, this research question is related to the overall project and has an evaluative character (project-related level). Looking toward future cross-cultural studies, we were interested in refining horizontal forms of knowledge production (research structure-related level) that consider culture-specific values, norms and societal structures (context-related level).

## Research Questions

As elaborated above, the research questions of this explorative interview study relate to three levels:

1. To the experiences of the Ecuadorian participants: How did they evaluate this specific project (the overall project) in terms of reciprocity and regarding their perception of the research processes, e.g., of data assessments (project-related level)?
2. To this specific cultural context: Which values, culture-specific concepts and societal structures are relevant (context-related level)?
3. To the role of reciprocal structures within research projects: How can research between equal partners succeed in this cultural context (structure-related level)?

We regarded these research questions as interrelated: For example, answers to the structure-related level were expected to depend on contextual or cultural specificities (context-related level). To address these questions, we drew on grounded theory (GT) (Corbin & Strauss, 2015); two of the authors conducted guided interviews with three groups of experts (study participants, Ecuadorian research assistants of the overall project, experts on research in Indigenous communities) that were then analyzed using thematic analysis (Braun & Clarke, 2006).

## Method

### Qualitative Research Design

All stages of this study were shaped from the social positions of the first authors, who are both White, German psychologists^ii^. For the study, we drew on qualitative methods because we were interested in approaching the field according to the principle of openness and in discovering new themes, giving credit to their complexity and interrelatedness. Specifically, we based our study on grounded theory (Corbin & Strauss, 2015), which implies that theoretical literature, reflections with those involved in the research project, and personal experiences in the research field give rise to constant adaptions (extending, refining, selecting) of the research questions, to the selection of interview partners and to approaches during data analysis.

### Participants

The overall project was approved by the scientific commission of the University of Otavalo. We informed the interviewees about the purpose of the interview study and about data handling (anonymization, further use of the data).

In line with the principle of selective sampling (Flick, 2021), we selected the following interview partners as experts for the three levels of interest (project-, context- and structure-related levels).

1. Four participants of the overall project (*study participants*; P01, P02, P03, P04)
2. Three Ecuadorian research assistants of the overall project (*research assistants*; A01, A02, A03):
  A01: Involved in the overall project since its beginning (data assessment, research coordination, recruiting participants); university degree in social and cultural development; identifies as Indigenous.
  A02: Former employee; one of the first contact persons for the German research team; involved in conceptual work, cultural mediator and piloting; university degree in economics; identifies as Indigenous.
  A03: Ecuadorian student assistant (studying social and cultural development) in the overall project; conducted weekly data assessments at the participating families’ homes together with German student assistants; identifies as Mestiza.
3. Three local researchers regarded as experts in conducting research in Indigenous communities in Ecuador (*experts on research in Indigenous communities*; R01, R02, R03):
  R01: Lecturer in global studies at the University Andina in Quito (Ecuador); in her research, she focuses on *buen vivir*^iii^; White^iv^.
  R02: Coordinator of the course Social and Cultural Development; psychologist; cooperation partner of the German research project at the local (Ecuadorian) university; conducts participatory research with the Chachi Indigenous group in Ecuador; Mestiza^iv^.
  R03: Lecturer in anthropology at the University of Otavalo; research focus on interculturality; Mestizo^iv^.

Four additional individuals were interviewed; their interviews were not considered further in the analysis.

## Data Collection

We used guided interviews with experts (Flick, 2021). Regarding the interviews’ degree of standardization, we used the guidelines as thematic frameworks, which provided impulses, but they were not considered mandatory for the course of the interview. The thematic focuses of the interviews are described below.

In total, ten open, guided interviews (Flick, 2021) were conducted and analyzed. The selection criteria for the three groups of interviewees were as follows: For selecting study participants, only those mothers who had participated in the workshops (one of the reciprocal elements) could be considered (*n* = 15), and, of those, several mothers could not be reached by phone; overall, the participating mothers were available and willing to participate in another interview. The Ecuadorian employees of the overall project were selected based on their long experience in the overall project. The experts on research in Indigenous communities were selected based on their experience regarding community-led research with Indigenous communities in Ecuador. Moreover, we included as many different perspectives as possible (with regard to social positions, professional backgrounds, research topics) with this study design.

Depending on the interviewee, different thematic focuses were set:

1. Study participants:
  - perceptions of reciprocity in the research project
  - subjective perceptions regarding the research processes (e.g., data assessments)
2. Research assistants:
  - subjective opinion and experiences regarding research processes in the overall project (e.g., What role does the foreignness of the researchers from Germany play? To what degree do they perceive the project as reciprocal?)
  - cultural context in Otavalo and Cotacachi
  - purpose of research
3. Experts on research in Indigenous communities:
  - research attitude and purpose of research
  - research ethics
  - societal structures/processes in the research field (R03)
  - *buen vivir* (R01)
  - evaluation of the overall project (R02)

Interviews with the employees and local researchers were conducted by one of the German authors in Spanish (*n* = 5) or German (*n* = 1). Interviews with study participants were conducted in Spanish (*n* = 3) or Kichwa (*n* = 1) by Nancy Beatriz Iza Simba – who, as our Indigenous colleague, is also one of the interviewees (A01).

### Thematic Analysis

The interviews were recorded and transcribed using the program f4 by one of the first authors. Next, she used MAXQDA software (VERBI, 2021) to conduct a thematic analysis (Braun & Clarke, 2006), which encompassed eight phases. The first phase was (1) becoming familiar with the data (e.g., through transcription): recording initial ideas about relevant themes and patterns and selecting ten interviews for further analysis (according to their relevance for the research questions).

The next phase involved (2) generating initial codes across the entire data set: At this stage, the abovementioned first author opted for two separate category systems, one for coding the study participants’ answers and one for coding the answers of the research assistants *and* the experts on research in Indigenous communities. The category systems were primarily developed inductively. Some additional codes were derived deductively in accordance with our research questions (values, participants’ motivation, reciprocity in research structures, cooperation with *comunidades*^v^), and the category *buen vivir* was derived from literature (Acosta, 2018; Huanacuni, 2010). A sentence was set as the smallest coding unit; also, parts of text could be assigned to multiple codes. For developing the category systems, we conducted a formative reliability test after three interviews, separately for both systems.

The next steps included (3) coding the remaining interviews and conducting summative reliability tests, (4) assigning the resulting codes to unifying themes and allocating themes to each research question (integration), (5) identifying the relationships between the codes, and (6) reviewing themes, generating thematic maps of the analysis encompassing main themes and sub-themes, and discussing those maps within collegial colloquia (with White, German psychologists) regarding their validity and discriminatory power. Next, (7) the specifics of each theme were refined, and all codes were integrated into one overarching thematic map. Themes that proved irrelevant to the research questions were not considered in the final phase. In the last phase, (8) results were produced, e.g., by selecting vivid quotes relating back to the research questions and literature. Throughout all phases, research questions were further refined.

## Results

**Figure 1.**
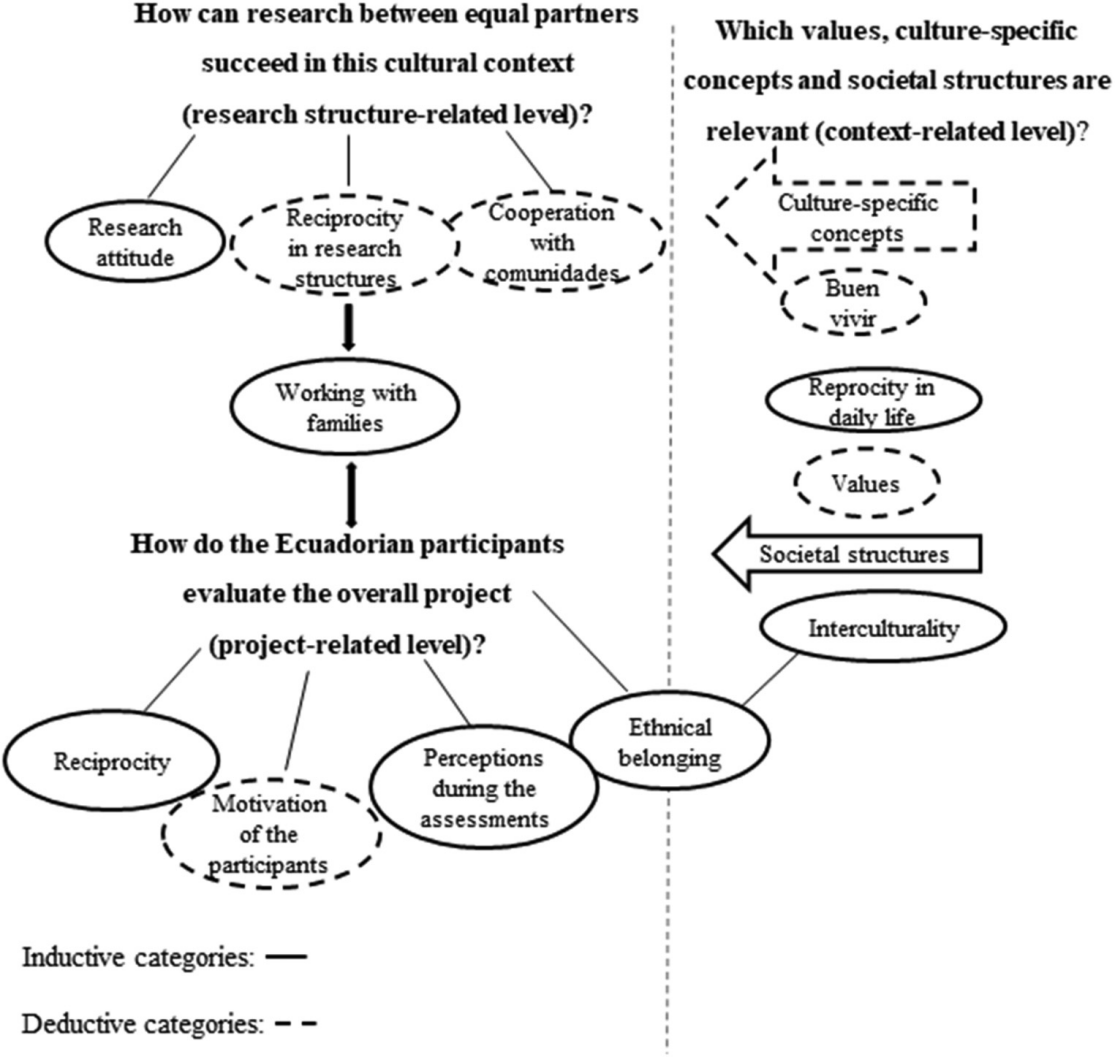
Main themes in relation to the research questions and to each other.

Here we present the main themes and sub-themes identified in the interviews, where sub-themes serve as the subordinate categories to or the concrete manifestation of main themes (for the overarching thematic map see Figure 1).

### Project-Related Level

The project-related level refers to the perceptions of the Kichwa mothers who participated in the overall project.

During the initial stages of the overall project, what was the mothers’ motivation to participate?

#### Motivation of Participants

Mothers mentioned three aspects that motivated them to participate: helping the research team, learning (from the study) and being curious (about the study).

#### Participants’ Perceptions During the Assessments

This category summarizes, in five subcategories, the mothers’ personal feelings during the assessments.

##### Positive Feelings

All interviewed mothers reported that they would participate in a similar study again. When asked by the interviewer why she would participate again, one mother answered, “Because, I don't know. It's something nice. To be with you guys. It's something nice, I don't know. It caught my attention” (P03: 62 - 65).

##### Negative Feelings

Yet, all the mothers reported feeling nervous during the assessments:

With the cameras, on the other hand, a bit, so then comes the nervousness with the camera and with the baby. So yes, I felt a bit nervous in front of the camera. Without the cameras, on the other hand, everything is calm, everything is normal. (P03: 37 - 37)

Shame. In the interview with the Kichwa-speaking study participant, it was clear that the mother felt ashamed when the interaction between her and her child was videotaped. She expressed insecurity about her behavior and performance: “From my point of view, the recordings were good and I thought it was good. But I also thought that maybe it was not good and I was ashamed of that” (P04: 92-92).

##### Attributed Feelings of the Infants

Some mothers surmised that their children also felt comfortable during the assessments: “When you were recording, sometimes I think she was also waiting a bit for them to come” (P01: 44-44).

##### Acclimatization Period

All mothers said that it took them some time to get used to the new situation. One mother commented, “MH, in the beginning I said: Mh, how? You're coming to visit me? And then I got used to it and yes, it seemed good” (P02: 49 - 49).

##### Trust

Two participants reported that they came to trust the research team; one mother expressed that she “grew to like them” (P03: 15-15) and another remembered that “then, they came to our house and we started to trust and we also felt good that they came to visit us” (P01: 50 - 50).

#### Reciprocity to Mothers

The immaterial rewards that mothers mentioned they gained from participating can be divided into five subcategories: friendship, learning experience, time/exchange with children, visit and information.

##### Friendship

“What I received was affection. Friendship and I think also honesty” (P03: 59 - 59).

##### Learning Experience

When asked whether she would participate in a similar study again, one participant answered, “To continue learning, I say yes” (P02: 74 - 75).

##### Time/Exchange with Children

One mother remembers particularly nice moments during the visits: “Yes, when I was taken in with my daughter. I was taken in with my daughter and talked to her when she was very small. Something I almost didn't do with my other daughter” (P03: 16 - 17).

##### Visit

Participants perceived the visits by the research team as a return for their contribution. One mother expressed that she received *“*company for some time” (P03: 55-55).

##### Information

One participant expressed that the “conversation” during the visits helped her and remembered that they (the research team) “gave me information on how to deal with my daughter” (P01: 32 - 32).

#### Ethnical Belonging

##### Role of White Persons in the Overall Project

The mothers evaluated the German students’ participation positively and did not attribute any particular influence to it. One participant explained that for them as a family, even with the presence of the Germans, “everything was normal” (P01: 55-56). It had not changed the mood, as they got along well.

### Context-Related Level

To more deeply understand this specific cultural context, we analyzed the interviews with two groups of experts (research assistants, experts on research in Indigenous communities).

#### Values

The respondents named hospitality, reliability, honesty, cohesion, generosity and reciprocity as the most important values in the *comunidad*.

As exemplified in the following quote, giving and receiving form an important basis of the collectivist structures within the Indigenous community: “Because when someone gives something, you have to give back twice as much” (A01: 26-26). Examples given by two research assistants are help from others (A01, A02), doing work for the *comunidad* collectively in the form of the *minga*^vi^ and the bond within the family (A02).

#### Reciprocity in Daily Life

Reciprocity emerged as an important cultural factor that is rooted in everyday life of the Indigenous communities. For instance, two assistants emphasized that part of their identity as Indigenous people is to never enter a house empty-handed, such that they always bring a gift when visiting relatives (A02) and always give the best of themselves (A01).

#### Buen Vivir

According to one local researcher (R01: 17-17), *buen vivir* is the “expression of a way of life” in which accumulation and imbalance are bad and are regulated by mechanisms inherent in the community. The statements in this category were largely congruent with those of reciprocity in everyday life. However, some statements related directly to the general concept of *buen vivir*, for example, that material goods exceeding one's own needs are to be shared communally (R01) and that a reciprocal relationship exists between people and nature (R01). One research assistant (A02) explained that – although this concept is diminishing – it is still lived in an energetic, spiritual and productive way. Yet, this way of life was not called *buen vivir* by the members of the *comunidad*, because it is overlaid by the political discourse about the concept.

Overall, of the most important values in the Indigenous communities, reciprocity is especially salient because it has a formative influence on individual and social life in these Indigenous communities.

Regarding societal structures that are relevant in this specific context, the interviewees especially emphasized interculturality.

#### Interculturality

According to one local researcher, the colonial period left its mark on society in and around Cotacachi and Otavalo. Today, “Otavalo [is] a sector with great cultural diversity” (R03: 6-6), where people from different ethnic backgrounds share the living space.

##### Ethnical Belonging

As evidenced in the following quote, the relationship between the different ethnic groups have been characterized by verticality:

Unfortunately, since colonization in Ecuador, there have been vertical relationships between Indigenous persons and Mestizos and white persons, with the Mestizos and the whites at the top and the Indigenous persons at the bottom. And the black persons much further down. The challenge and the importance of interculturality is to try to break through these structures that are very much at the center here in Ecuador, racist structures. Interculturality is another way of building relationships between us who live together. (R03: 6 - 6)

The experts reported a “historical mistrust” (R02: 30-30) of the Indigenous population toward the Mestizxs (for example, from the times of the *haciendas*^vii^) and also mentioned current structures of racism, rivalry and inequality (A03). Although opportunities for Indigenxs and Mestizxs are slowly equalizing, discrimination still exists in public spaces, such as in school (A01).

##### Ser Indigena

As a sub-theme, this category specifies one manifestation of interculturality, specifically regarding what it means to be Indigenous and in what ways societal processes have influenced the perception of this ethnic identity. Although one assistant (A02) described that discovering what it means to be Indigenous is a very individual process (A02), the respondent stated that they share an “environment of activities or traditions” (A01: 16-16), a culture, worldview, and knowledge (A02). As such, Indigenxs are also obliged to pass on the culture and live it with pride.

As explained above, the Indigenous community in and around Cotacachi/Otavalo is united by the experience of years of discrimination and marginalization within society and the common, organized rebellion against it (A02):

And here in Otavalo too, because we didn't all have access to education and we didn't all have the possibility to go by bus or to go into a restaurant (…). They wouldn't let us in, those were places for Mestizos. (A01: 20 - 20)

This implies that cohesion within the community is highly valued and that a common language and identity “opens doors” (A01: 16-16).

Overall, the main theme interculturality shows that ethnic identity and the associated historical and current experiences shape social life in and around Cotacachi and Otavalo. Especially relevant are the tensions between Mestizxs and Indigenxs and the confrontation with colonial history and more recent dependency relations; these aspects seem fundamental for researchers to understand before preparing future research projects intended to occur in this cultural context.

### Structure-Related Level

Based on the interviews with research assistants and experts on research in Indigenous communities, four main themes were identified: research attitude, reciprocity in research structures, working with families and cooperation with *comunidades*. That is, the research question on how research between equal partners can succeed in *this* cultural context relates to subordinate aspects as well as the concrete working level.

#### Research Attitude

Several experts stressed that the basis for research between equal partners is created by the research attitude, which manifests via the three following sub-themes:

##### Research Purpose

The interviewees indicated that research should benefit society and its results should be applied in practice. This implies that research topics and the design of research projects should be oriented toward the needs of the researched:

**“**And the thing is that *they* learn from it in the first place, not that I conduct research” (R01:25-25).

The experts criticized research that only serves the academic careers of individuals. This idea was accompanied by questioning the (personal and scientific) motivation behind research.

##### Personal Attitude

Behind every research project are people who shape it and make relevant decisions. These resulting structures shape the configuration of research relationships:

No. It's a question of attitude. I like to do it that way. I think it's the right thing to do because I also believe that the university has to contribute to society. And that it is not enough to publish in some journal or other. (R01: 29-29)

##### Research Design

One indicator of the pursuit of an equal research relationship can be the choice of a specific research design. e.g., by planning the research process *with* the researched (R02: 10-10).

#### Reciprocity in Research Structures

According to the experts, the researchers’ agency also includes decisions about the extent to which reciprocity is considered in research processes and structures. In this context, the transfer of knowledge was identified as a relevant level on which a balance between participants and researchers can be established:

##### Transfer of Knowledge

An important component is disseminating the results to the researched. One local researcher commented:

“It depends. So mostly, even when I'm done with my research, I go back and share what I've found out” (R01: 25-25). This includes not only presenting the results, but also entering into dialogue about them.

Importantly, knowledge transfer as a form of reciprocity also involves “relating (to) local knowledge” (R01: 53-53). Moreover, reciprocity in the research process can be sought through material compensation and the involvement of local, trained employees.

#### Working with Families

This main theme links the structure-related and the project-related levels (see Figure 1). Four sub-themes spell out respectful behavior when conducting research with Kichwa families:

##### Behavioral Norms

These norms include friendliness, politeness and respecting the families’ rhythm of life.

##### Time and Patience

One research assistant commented that it is essential to be flexible during the visits, to be patient and not to be intrusive:

So that's how it is and I can't say it's frustrating for me when a family is not there. Because I said that the family is doing us a big favor by hosting us and we cannot overdo it. Rather, we have to be flexible with them. (A01: 42-42)

##### Transparency

According to one assistant, being honest and open builds trust:

So in the beginning they can be scared, we can be scared, but that's until they know what it's about and after that it's nice - so you walk through that door of trust and you become part of the family. (A01: 34 - 34)

##### Trust Through the Same Ethnicity

In the case of the overall project, it was particularly helpful to involve Indigenous research assistants, making it easier to achieve an initial familiarity and a sense of security:

But still, the very fact that you are Indigenous helps a lot and opens many doors. Just the fact of speaking in Kichwa is something that gives them security and they know that they are dealing with a person who knows what they are. (A01: 16 - 16)

#### Cooperation with *Comunidades*

Next, the main theme cooperating with *comunidades* with respect manifests in the following ways:

##### Get to Know the *Comunidad*

One research assistant pointed out that during the initial stages of the research project, “you have to get to know a bit of the reality, of what the *comunidades* are like, the people and so on” (A02: 2-2).

##### Authorization

One interviewee stated that one “should (…) always respect the hierarchical order of authorities within the *comunidad*.” (A01: 6-6). This “facilitates basic steps” (A01: 6-6) and guarantees support if complications arise in the research process.

##### Contact

When using networks and intermediaries between the *comunidad* and the research team, the social responsibility for the intermediaries also increases, because they give their word, so to speak, and stand up for the project as a person:

And sure, in this part I spoke with [anonymized community leader]. He knows where I'm coming from. And clearly, if the research had gone badly, then I would have looked bad and in the *comunidad* they would have talked to me about it. (A02: 8-8)

## Discussion

### Project-Related Level

Regarding the experiences of the Ecuadorian participants, the mothers generally evaluated the project positively; for example, they were glad about the visits by the research team, about established friendships and about the learning experience. One interpretation of the generally positive feedback is that at the structural (e.g., reciprocal elements) and/or the individual level (e.g., to keep them company), culture-specific values were considered and formatively influenced the experiences that mothers had during the assessments and/or during the workshops. Alternative (or additional) interpretations are that mainly those mothers who positively viewed the research project agreed to participate or that the interviewed mothers were afraid to mention critical aspects because they shared close relationships with the interviewer and did not want to offend her or because she was associated with the German research team. The reason we chose the Indigenous colleague as an interviewer was to ensure a greater level of trust during the interviews. For future studies, we recommend choosing a person who the participants are familiar with (e.g., from the same community), who shares the same ethnicity with the participants and who does not hold a position within the research team.

During the weekly visits, spontaneous interactions between the Kichwa mothers and their babies were videotaped by bicultural teams of German and Ecuadorian assistants, a setting that evoked negative feeling in mothers too: Nervousness was mentioned by some mothers, and the (only) Kichwa-speaking mother, being afraid of being evaluated negatively by the observer, also experienced feeling shame. One possible interpretation to investigate further is that the mother's perception of her ethnic or cultural inferiority, which may be historically rooted, evoked those feelings of shame, a specific mindset that has been coined *colonial mentality* (David & Okazaki, 2010).

Numerous authors have recently pointed to the ethical challenges of cross-cultural research projects. While sharing this concern, we argue that it is important to talk directly to study participants about their perceptions. Ideally, the formative and summative evaluation of the study participants’ perceptions (e.g., regarding the research process, the consideration of cultural norms, possible reproduction of power asymmetries) would become an elementary part of cross-cultural projects.

### Context-Related Level

The experts named hospitality, reliability, honesty, cohesion and generosity as fundamental pillars of the Indigenous culture. Moreover, the central importance of reciprocity – that is, the balance between giving and receiving, by means of which *buen vivir* manifests in everyday life – was accentuated. According to two experts, sharing these common values and associated traditions forms a key aspect of Indigenous identity and sense of community; it follows that these values inform behavioral norms in Indigenous communities. Furthermore, social life in and around Otavalo and Cotacachi is strongly influenced by its interculturality, especially by the relationships between Indigenxs and Mestizxs.

Overall, it is important to contextualize the positions of the experts, as they unfold their meaning amid the larger context of culture-specific concepts like the *cosmovision andina* (Andean Wordview), encompassing the belief that all elements of the cosmos are interconnected and should stand in dialogue to each other (Mamani Choque & Ramos Alcalá, 2013), and the concept of *buen vivir* (Acosta, 2018), which is part of the *cosmovision andina* and defines a way of life that is in balance with nature and other humans. The shared colonial past as well as current experiences of discrimination, such as with respect to schooling, healthcare and career opportunities (Masala & Monni, 2019), also serve as interpretational contexts for the findings.

Many of the identified culture-specific values, concepts and structures are consistent with literature. Yet, the empirical approach to the context-related level allows for a deeper understanding of the complexity (e.g., perceived tensions between Mestizxs and Indigenxs in everyday life), the interrelatedness (e.g., between *buen vivir* and reciprocity), and the culture-historical embeddedness of the themes (e.g., rebellion of the Indigenous community against discrimination and, today, the common identity “opens doors”); these facets are crucially important for organizing and designing research projects in this specific context.

### Structure-Related Level

In the following, we focus our discussion of the structure-related level on the main themes research attitude and reciprocity in research structures before discussing direct implications for cross-cultural developmental psychology projects: Regarding the question of how research between equal partners can succeed in *this* cultural context, the experts highlighted the attitude around the purpose of research and the personal attitudes of the researchers; both factors relate to decisions about a specific research design: If the researcher is motivated to do research based on partnership and stands for common-benefit research, decisions regarding the research design can be a first step toward reducing hierarchies.

Moreover, the experts stressed enlarging the degree of reciprocity in research structures; in this regard, they specifically emphasized entering into dialogue about the findings (knowledge transfer: learn from each other; see also Broesch et al., 2020) and taking into account local knowledge.

We now discuss how the abovementioned factors regarding research between equal partners can be applied to the field of cross-cultural developmental psychology. More specifically, none of the interviewed experts conducts developmental psychology research, namely basic scientific research that, by definition and independent of personal attitudes, generates academic knowledge that creates the basis for decisions or interventions that benefit society (and the participants) but is not justified by directly doing so. However, as a promising avenue for equitable research, the experts stressed two entangled aspects that may have direct implications for cross-cultural developmental psychology: First, they advocate for considering other forms of knowledge and, second, they stress the *how* of knowledge production. Using the example of the overall project, the German research team and a local research team could have discussed the commonalities and differences in conceptions about child development. Questions like the following could have been addressed: To what extent does the Andean worldview affect your conception of child development? What parental ethnotheories are especially relevant shortly after the birth of a child? How active is the role that mothers attribute to themselves, for example, regarding the coregulation of infant states? Building on this dialogue, research questions and measures could have been developed cooperatively.

Some disciplines within the social sciences are already successfully integrating different systems of knowledge (e.g., theoretical foundations from the Global North and the Global South; scientific and non-academic knowledge; quantitative and qualitative methodologies; Center for Advanced Latin American Studies, 2017). However, (Euro-American) psychological science still holds a special position, one that is characterized by distinction from other systems of knowledge rather than by their mutual enrichment and by the entanglement of ethical and epistemological research questions (What knowledge is regarded as knowledge?).

In accordance with Marfo (2016), we regard the diversification of our databases as “only the first step” (p. 180) toward a more global science of human development. As our field “remains largely entrapped in Euro-American conceptions of development” (p. 180), calls remain for “increased openness to, and deliberate advancement of, context-analytic research exploring variations and similarities in developmental constructs across cultures” (p. 180).

On a political level, we welcome incentives for developmental psychologists from the Global North to expand their degree of cooperation with partners in the Global South. For example, funders of cross-cultural research projects could include collaboratively elaborated project proposals as an eligibility criterion.

### Integration of the Results

Figure 1 demonstrates the main themes at the project-, context- and structure-related level and demonstrates how the categories within the three levels are interrelated: Dependent on the values and societal structures in a specific context, the categories and manifestations at the other two levels change. For example, the extent to which the category *ethnical belonging* affects the experiences of study participants or should affect the organization/design of the research project is modulated by societal structures in the field, such as interculturality.

### Limitations of the Present Study

In this study, we used the example of a psychological research project in the Andean context to explore ethical aspects of cross-cultural projects at three interrelated levels. The research design and methods (qualitative design, grounded theory approach, relatively unstandardized interview guideline, thematic analysis of the data) gave credit to the heterogeneity and complexity of the research questions and allowed for flexibility during the research process and for exploring novel themes. Due to the paucity of empirical studies addressing ethical challenges of intercultural research, our analytic approach focused on giving an overall description of relevant themes. In doing so, some complexity (e.g., patterns exceeding the structure of main themes and sub-themes) was lost, making it difficult to determine the *keyness* of a theme (Braun & Clarke, 2006). Moreover, the samples for each research question were too small to achieve saturation, limiting the interpretability of our findings. A critical aspect regarding the codings is that some categories had content overlap (e.g., personal attitude vs. research attitude) and, therefore, were not highly selective (criterion of distinctiveness of categories not fully met; see Braun and Clarke, 2006, p. 96). In future studies, high (ecological) validity of the codings might best be achieved by conducting interrater reliabilities in intercultural teams of researchers. Importantly, despite the critique of the authors regarding vertical research relationships, the Global North perspective significantly influenced all stages of this interview study, and our research questions were not investigated in a participatory way.

### Best Practices

At the research-structural level, establishing horizontal research relationships between researchers and the researched as well as between German (i.e., WEIRD) researchers and the local Ecuadorian research team, such as by using a participatory research design, allows for the best adaption to context-specific values (e.g., balance between giving and receiving), norms and societal structures (context-related level). This recommendation generalizes to research relationships characterized by strong power imbalances.

### Research Agenda

Here, we outline different avenues for future research. Regarding the experiences of participants in cross-cultural studies in the Global South, future studies should aim at including the whole sample of participants and might benefit from using both focus groups (to encourage participants to talk freely) and more standardized interviews. Regarding the context-related level, the broad exploration of values, culture-specific concepts and societal structures could be combined with more in-depth (e.g., about *the most central* values) and theory-driven questions and analyses (e.g., How do values affect behavior?). For example, this approach might help dissolve the entanglement between the value of reciprocity, reciprocity in everyday life and *buen vivir* and increase the complexity of the findings. At the structure-related level, it would be promising to interview experts, ideally developmental psychologists, with experience in successfully integrating quantitative research methodology and Euro-American conceptions of development with other systems of knowledge (e.g., How can quality criteria be diversified?). Another promising avenue is to take an organizational behavior and team development view on cross-cultural research projects and to specify what *cultural synergy* – that is, the capacity to use cultural diversity as a resource – could mean in those projects (Adler, 2008): Along with possible indicators of the degree of cultural synergy identified here (e.g., in how far have research questions and measures been developed cooperatively?), additional factors, which have an influence on cooperation in intercultural teams, such as intercultural competence, tolerance and flexibility of team members, could be addressed in future studies.

Moreover, future studies should evaluate whether feelings associated with perceptions of inferiority are frequently activated in the context of cross-cultural studies in the Global South (or are activated more frequently than during the same assessments in the Global North) and should explore possible associations between colonial mentality and feelings of shame.

### Educational Implications

**Figure 2.**
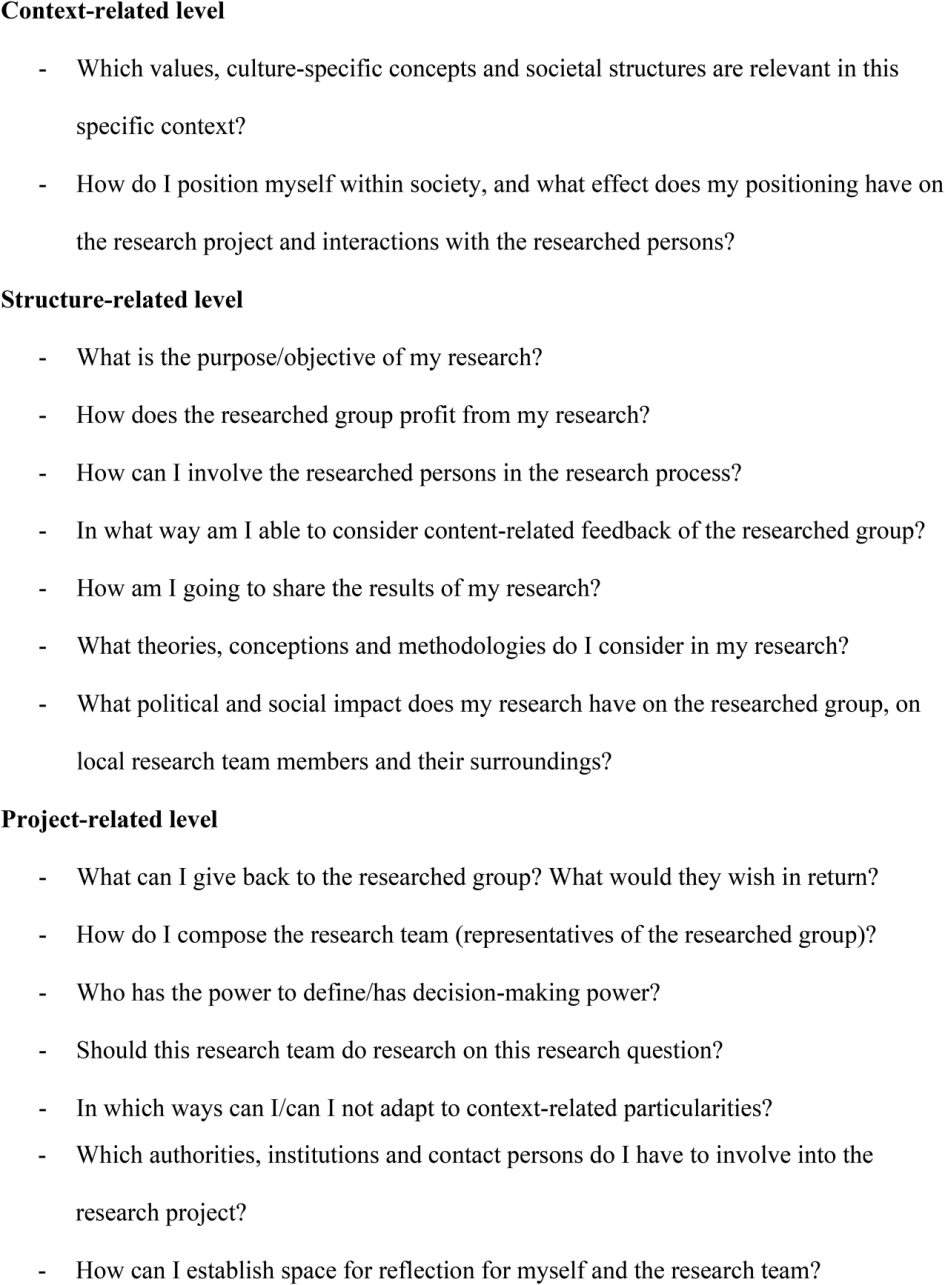
Reflective questions for designing a research relationship based on equal partnership.

Based on the thematic analysis results (see Figure 1), we derived self-reflective questions that researchers who are motivated to design research relationships based on equal partnership can use when preparing future research projects (see Figure 2). Furthermore, joint trainings with the whole intercultural research team that consider the cultural background of participants and power asymmetries could create the basis for forming researchers’ personal attitudes and for informed decisions on a specific research design.

## Conclusion

Despite the current debate about ethical challenges of psychological cross-cultural research projects, few evaluation studies have addressed the actual experiences of participants and there is an paucity of empirically grounded, context-specific solutions to those challenges (e.g., regarding research design and processes). Against this background, this explorative interview study evaluated the experiences of Kichwa mothers who participated in a developmental psychology research project on early mother-infant interaction and, via interviews with two additional groups of experts, addressed the question of how research between equal partners can succeed in *this* cultural context. Overall, study participants’ perceptions were multifaceted (e.g., valuing established friendships vs. expressing feelings of shame). Moreover, the interview analysis identified that establishing horizontal research relationships allows for the best adaption to context-specific values (e.g., balance between giving and receiving), norms and societal structures. Regarding applying the findings to the field of cross-cultural developmental psychology, we mention two aspects, namely the *how* of knowledge production and the integration of different systems of knowledge, that play a pivotal role when power equality is the objective. In line with others, we argue that diversifying the empirical databases is only the first step toward a more global science of human development and propose that the next step is diversifying our scientific perspectives.

In view of future cross-cultural studies, we call for more resources (time and financial) dedicated to reflecting on the unique ethical challenges of those projects.

## Notes

1. For brevity, we refer to those cross-cultural research projects as *cross-cultural*.

2. See Authors’ Biographical Sketches for further information on researcher characteristics and reflexivity as related to this study.

3. *Buen vivir* is a collectivist concept of society based on Indigenous ideas and way of life and encompasses the unison between people and nature as well as degrowth thoughts.

4. This is an external ascription; we did not explicitly ask for social positioning.

5. *Comunidades* refers to the collective form of living in the field site and structurally corresponds to a village. It also corresponds to the community of the Indigenous rural population, which is organized according to certain hierarchies and which – historically – has had a marginalized position within society.

6. *Minga* refers to solidary work that serves the common good. This work both secures basic care and serves the community as such. Examples are collaboratively built streets or schools and the preparation of festivities or agricultural works.

7. During colonization, many people were expropriated from their land, which was given to the conquistadors. Those areas often gave rise to *haciendas*, which were estates where Indigenous people lived under strong dependencies and received a piece of land in return for their work.
